# High hypoxia status in pancreatic cancer is associated with multiple hallmarks of an immunosuppressive tumor microenvironment

**DOI:** 10.3389/fimmu.2024.1360629

**Published:** 2024-03-06

**Authors:** Hassan Sadozai, Animesh Acharjee, Hateem Z. Kayani, Thomas Gruber, Reginald M. Gorczynski, Bernard Burke

**Affiliations:** ^1^ Centre for Health and Life Sciences, Coventry University, Coventry, United Kingdom; ^2^ Institute of Cancer and Genomic Sciences, University of Birmingham, Birmingham, United Kingdom; ^3^ Independent Scholar, National Coalition of Independent Scholars, Visp, Switzerland; ^4^ Institute of Medical Sciences, University of Toronto, Toronto, ON, Canada

**Keywords:** hypoxia, tumor microenvironment (TME), pancreatic ductal adenocarcinoma (PDAC), immune checkpoint, galectins

## Abstract

**Introduction:**

Pancreatic ductal adenocarcinoma (PDAC), the most common form of pancreatic cancer, is a particularly lethal disease that is often diagnosed late and is refractory to most forms of treatment. Tumour hypoxia is a key hallmark of PDAC and is purported to contribute to multiple facets of disease progression such as treatment resistance, increased invasiveness, metabolic reprogramming, and immunosuppression.

**Methods:**

We used the Buffa gene signature as a hypoxia score to profile transcriptomics datasets from PDAC cases. We performed cell-type deconvolution and gene expression profiling approaches to compare the immunological phenotypes of cases with low and high hypoxia scores. We further supported our findings by qPCR analyses in PDAC cell lines cultured in hypoxic conditions.

**Results:**

First, we demonstrated that this hypoxia score is associated with increased tumour grade and reduced survival suggesting that this score is correlated to disease progression. Subsequently, we compared the immune phenotypes of cases with high versus low hypoxia score expression (Hypoxia^HI^ vs. Hypoxia^LOW^) to show that high hypoxia is associated with reduced levels of T cells, NK cells and dendritic cells (DC), including the crucial cDC1 subset. Concomitantly, immune-related gene expression profiling revealed that compared to Hypoxia^LOW^ tumours, mRNA levels for multiple immunosuppressive molecules were notably elevated in Hypoxia^HI^ cases. Using a Random Forest machine learning approach for variable selection, we identified *LGALS3* (Galectin-3) as the top gene associated with high hypoxia status and confirmed its expression in hypoxic PDAC cell lines.

**Discussion:**

In summary, we demonstrated novel associations between hypoxia and multiple immunosuppressive mediators in PDAC, highlighting avenues for improving PDAC immunotherapy by targeting these immune molecules in combination with hypoxia-targeted drugs.

## Introduction

Pancreatic ductal adenocarcinoma (PDAC), which constitutes approximately 90% of all pancreatic cancer cases, is a highly aggressive malignancy (1, 2). Notably, 80% of cases are diagnosed at a late stage, which precludes surgical resection, the only potential cure (2, 3). Currently, the use of polychemotherapy regimens such as mFOLFIRINOX and gemcitabine/nab-paclitaxel leads to only modest improvements in outcome and the 5-year survival rate is roughly 10% (2, 4). Furthermore, PDAC incidence is increasing in many countries in Europe and in the USA (5, 6). PDAC is primarily driven by mutations in 4 genes (*KRAS*, *TP53*, *CDKN2A* and *SMAD4*) and displays significant therapeutic resistance to both chemotherapies and radiation (7, 8). In the past decade, cancer immunotherapy, particularly immune checkpoint blockade with antibodies targeted to the checkpoint receptors CTLA-4 and PD-1, have shown remarkable response rates in some cancer types such as melanoma, non-small cell lung cancer and mismatch-repair deficient (dMMR) colorectal cancer (9). Conversely, PD-1 blockade was found to exhibit efficacy only in PDAC cases that are mismatch-repair deficient (10), which constitute only approximately 1% of all PDAC cases (11). Nevertheless, preclinical studies in mice, and some clinical trials have provided evidence that certain immunomodulatory treatments targeted to myeloid cells (e.g. CCR2 inhibitor) can lead to enhanced anti-tumor immunity in PDAC (12). Thus, there remains the possibility that additional research on the immune phenotypes of PDAC could yield novel immunotherapy-based approaches for treating this lethal disease.

A major barrier to effective treatment of PDAC is an exceptionally atypical tumor microenvironment (TME) marked by high levels of desmoplasia (fibrosis), poor vascularization and the abundance of multiple subsets of immunosuppressive myeloid cells such as myeloid-derived suppressor cells (MDSC) and tumor-associated macrophages (TAM) (13, 14). Furthermore, tumor hypoxia, which arises due to abnormal, insufficient vasculature, and enhanced oxygen demand by tumor and stromal cells, is a major pathological hallmark of PDAC (14, 15). The cellular response to hypoxia is primarily driven by the hypoxia-inducible factor (HIF) family of transcription factors namely HIF-1, HIF-2 and HIF-3, comprising an oxygen-sensitive HIF-α subunit which forms a dimer with the constitutively expressed beta subunit (16, 17). An early report demonstrated that HIF-1α was overexpressed in PDAC tissues and absent from non-malignant tissue as well as being associated with advanced disease stage (18). Moreover, multiple studies in the literature have documented that hypoxia modulates multiple facets of disease progression in cancer including genomic instability, EMT (epithelial-mesenchymal transition), metabolic reprogramming and immunosuppression (15–17). Targeting hypoxia to abolish immunosuppression is an exciting prospect for developing more effective treatments for PDAC. In a PDAC murine model, genetic deletion of HIF-2α, but not HIF-1α, in cancer-associated fibroblasts (CAFs), led to significantly delayed tumor growth (19). Moreover, these authors also observed that deletion of HIF-2α in CAFs also led to reduced intra-tumoral infiltration of M2 macrophages and regulatory T cells. However, further studies are warranted to dissect the immune landscape of hypoxic pancreatic adenocarcinomas. Recently, gene signatures for hypoxia have been used to study patient outcomes and characterize the TME using transcriptomics data from a number of cancers including gastric cancer (20), bladder cancer (21), and PDAC (22–24). One of the most widely utilized gene signatures for hypoxia is a 51-gene score developed Buffa et al. (25). Recently, this score was also shown to have prognostic utility in RNA sequencing (RNA-seq) cohorts of lung adenocarcinoma from The Cancer Genome Atlas (TCGA) (26) and was utilized to study hypoxia-related proteins in metastases from 17 distinct carcinomas in TCGA including PDAC (27).

In the present study, we used the Buffa signature, hereafter referred to as the hypoxia score, to profile PDAC cases from TCGA and compare the tumor immune microenvironments of cases with high versus low hypoxia score status. We demonstrated that high hypoxia scores are associated with a distinctly immunosuppressive TME and identified immune genes with a strong correlation to high hypoxia and confirmed these findings in a secondary cohort of RNA-seq data from PDAC cases.

## Materials and methods

### Cell culture

Human pancreatic cancer cell lines (BxPC3; CRL-1687, PANC-1; CRL-1469) were purchased from the American Tissue Culture Collection (ATCC, USA). PANC-1 cells were maintained in DMEM with 10% FBS, 100 U/ml penicillin, and 100 µg/ml streptomycin. BxPC3 cells were maintained in RPMI-1640 with 10% FBS, 100 U/ml penicillin, and 100 µg/ml streptomycin. PANC-1 (3x10^5^ cells per flask) or BxPC3 (1x10^6^ cells per flask) were seeded in 75cm^2^ cell culture flasks in 10ml of medium and were incubated under normoxic conditions (20.9% O_2_, 5% CO_2_) for 6 days or 4 days, respectively. The medium was then changed, and the cells incubated in normoxia for a further 24h before being cultured for a further 24h under either normoxic or hypoxic (0.5% O_2_, 5% CO_2_) conditions.

### RNA isolation and Real-time RT-PCR

RNA was isolated using Quick-RNA Miniprep Kit (ZymoResearch) according to the manufacturer’s instructions. Reverse transcription was carried out using a Tetro cDNA Synthesis kit (Bioline). Amplifications were carried out on a BioRad CFX Connect Real-time PCR machine (BioRad, England) using the following cycling parameters: initial denaturation at 95°C for 5 min then 40 cycles of 95°C for 10 seconds, 60°C for 15 seconds, 72°C for 30 seconds. PCR data were normalized to the relative amount of β2M housekeeping gene mRNA determined by separate PCR on each sample. The primer pairs used for each gene are shown in **Supplementary Table 1**. 

### TCGA dataset and hypoxia score assessment

Multiple transcriptomics datasets were utilized in this study. For all datasets utilized in this study, no exclusion criteria were applied on the basis of clinical variables. All patients with available gene expression profiles and curated survival data were included in the study. We downloaded uniformly processed gene expression data for TCGA, TARGET and GTEx studies from UCSC Xena Browser (Accessed on 28 September 2020). The analyses in this manuscript were limited to the samples from TCGA PAAD (PDAC) cohort. Sample metadata, including previously curated clinical data (28), were also obtained from Xena Browser. We computed hypoxia scores using the 51-gene signature described by Buffa and colleagues (25). We reviewed the signature to ensure correct mapping to the TCGA expression data gene annotation. The score was computed across all the samples in TCGA PAAD cohort using the rank-based, single sample scoring method implemented in the singscore package (29), with log2-transformed TPM values as input for expression measurement. For the TCGA PAAD cohort, we identified 176 primary tumor samples for which survival outcomes and expression values were available in both TPM (transcripts per million) and RSEM (RNA-seq by Expectation Maximization) expected count formats. The samples in the bottom and top quartiles of hypoxia score were designated as Hypoxia^LOW^ and Hypoxia^HI^, respectively (n=44 each). These cohorts were used to contrast samples with high and low levels of hypoxia throughout this manuscript, unless otherwise stated.

### Survival analysis

Association between overall survival (OS) and hypoxia in the PAAD cohort including sex, age at diagnosis, tumor stage and histological grade as covariates were evaluated within a Cox Proportional Hazard regression model using survival package in R (30, 31). For this analysis, 5 samples with missing stage and grade annotations were excluded leaving 171 samples for the analysis. Granular tumor stages were collapsed into their parent categories (e.g. Stage IA and IB into Stage I). Due to low representation of advanced stages in the dataset, Stages III and IV were collapsed into a single level Stage III/IV. Similarly, samples with histological grade G3 and G4 were combined into a single cohort of G3/4. For this analysis, hypoxia was modelled as a continuous hypoxia score.

To visualise differences in OS and progression-free survival (PFS) between high and low hypoxia cohorts, we generated Kaplan-Meier plots using survminer package (32), contrasting survival curves of patients in the top and bottom quartiles of the hypoxia score. The p values shown on the Kaplan-Meier plots were derived by Log Rank test comparing survival between these two cohorts (30, 31).

### Differential gene expression analysis

Differential expression analysis of genes between high and low hypoxia samples was performed using the edgeR package (33). To obtain raw counts, the RSEM expected count matrix obtained from Xena Browser was anti-log2 transformed and the prior count of 1 was subtracted (Accessed on 19 March 2023). To remove lowly expressed genes, the average gene-level log2 counts per million (CPM) values were computed across all samples in the expression matrix. Genes with average log2 CPM value below zero were removed, leaving 17214 genes for subsequent differential expression analysis. We computed scaling normalisation factors using trimmed mean of M values (TMM) method (34). Post-normalisation, we subsetted the expression dataset to samples in Hypoxia^LOW^ and Hypoxia^HI^ groups based on the bottom and the top quartiles of the hypoxia score (n=44 each). The significance of expression difference between the two cohorts was assessed for all of 17214 genes using a quasi-likelihood F test framework following the edgeR package user guide. The gene-wise p value was adjusted for multiple testing using Benjamini-Hochberg method (35). All subsequent references herein regarding genes differentially expressed between high and low hypoxia samples in TCGA dataset refer to this adjusted p value.

### Estimating TME cell-type abundance

Cell abundance estimate was performed using Microenvironment Cell Populations-counter (MCP-counter tool) (36), as implemented in immunedeconv package (37). Anti-log2 transformed TPM matrix was used as the gene expression input. To restrict expression table to uniquely annotated genes, we removed rows with duplicated gene symbols keeping the entry with the highest standard deviation.

Statistical comparisons in immune cell frequency between high and low hypoxia groups were performed using Mann-Whitney U tests.

Figures and text referencing differences in cell frequencies report p values adjusted using the Benjamini-Hochberg method (35). Signature scores for cDC1 were computed using the singscore package (29). Log2-transformed TPM matrix was used as gene expression input and the following cDC1 signature from a recent publication: *CLEC9A*, *XCR1*, *CLNK* and *BATF3* (38) was selected. Association between the hypoxia and cDC1 activation score was assessed by contrasting samples within top and bottom quartiles of the hypoxia score using a Mann-Whitney U test (Hypoxia^HI^ vs Hypoxia^LOW^).

### Analysis of hypoxia between PDAC and normal pancreas tissue

To compare hypoxia levels between tumor and normal pancreas tissue, a previously published dataset was utilized (39). The expression matrix, sample metadata and gene annotation tables were downloaded from GEO (GSE28735). The expression matrix was quantile-normalised using the affyPLM package (40), and log2-transformed. The resulting matrix was used as input to the singscore package (29), to compute hypoxia scores. The difference in hypoxia scores between matched tumor and normal samples was assessed using a paired Mann-Whitney U test.

### Random forest, feature elimination process and validation in additional cohort

Random Forest (41), a machine learning ensemble method was used to find the immune gene, the expression level of which could best identify the difference between Hypoxia^HI^ vs. Hypoxia^LOW^ tumors. Random Forest uses a bootstrapping method to pick random samples from the dataset. The samples are further split into training samples (two thirds of the set) and testing samples (one third of the set). The testing samples, which are also known as “out-of-bag” samples, are used to determine the accuracy of future predictions. This approach warrants that the operator set the number of trees (ntree) and the number of genes that are randomly chosen as options at each split for the Random Forest model to work. The ntree value was set to 500, and the mtry value was the square root of the factors.

To select the best genes that predict Hypoxia^HI^ vs Hypoxia^LOW^ tumors, iterative fitting to create random forests was utilized. With each iteration, a new forest was begun by removing 20% of the factors that were the least important. The set of variables that was chosen is used to predict how well the model will fit so that the “out-of-bag” error rate can be checked. The varSelRF function from the varSelRF package in R was used to perform this recursive feature elimination method process (41, 42).

The 5 genes retained in the final model were validated for their association with hypoxia in an independent RNA-seq dataset of 51 pancreatic adenocarcinoma samples (GSE79668). Pre-processed gene-level counts and samples metadata from GEO were downloaded. A filtering threshold was applied for lowly expressed genes by removing genes with average log2 count per million below value of 0, while ensuring that all genes from Buffa signature were retained. Normalisation factors were calculated using the TMM method (34), and computed log2-transformed count per million (CPM) matrix with a prior count of 1. The resulting log2 CPM matrix was used as input into the singscore method to calculate hypoxia score and as the measure of gene expression to perform correlation analysis between each of the genes and the hypoxia score.

### Statistical analyses

Statistical analyses were performed using R v3.6.0 or GraphPad Prism v10. The statistical tests used to compare data are presented in the figure legends. Where data are shown as boxplots, the box represents the IQR, and the whiskers extend 1.5x IQR below and above bottom and top quartiles respectively. A value of p<0.05 was deemed to be statistically significant. Low p values were reported as p<0.001.

## Results

### Assessment of the Buffa hypoxia score for PDAC profiling

**Figure 1:**
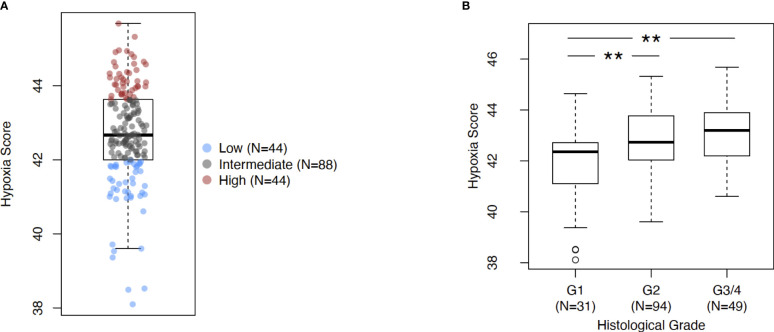
Assessing Buffa signature score in PDAC. **(A)** Box plot of sample-level hypoxia scores across the cohort of PDAC cases in TCGA. Samples with the hypoxia score in top and bottom quartiles were assigned into Hypoxia^HI^ and Hypoxia^LOW^ cohorts respectively. Each dot represents a single sample. **(B)** Box plots comparing hypoxia score distribution across reported histological grades. Statistical significance was determined using pair-wise Mann-Whitney U tests between groups and Holm-Sidak multiple testing correction was performed. ** p<0.01.

The investigations performed in this study are depicted as a flow chart in **Supplementary Figure 1**. First, we profiled the expression levels of the previously developed hypoxia score in pancreatic cancer (**Figure 1**). Using a rank-based gene signature scoring method for single samples (singscore) (29), we profiled 176 PDAC cases from the TCGA cohort for hypoxia score expression (**Figure 1A**). Next, we examined the expression of the hypoxia score across histological tumor grades. Higher tumor grades represent increased anaplasia and disordered structure and are generally associated with aggressive disease (43). As represented in **Figure 1B**, we observed a significant increase in the hypoxia score across tumor grades from Grade 1 to Grade 2 (p=0.003) and from Grade 2 to Grade 3/4 (p=0.003) (Grades 3 and 4 were collapsed into a single category due to there being so few cases of Grade 4 in our cohort). Here, we demonstrate that PDAC cases display varying levels of hypoxia score expression but also that the hypoxia score is increased in higher grade tumors suggesting that hypoxia is associated with aggressive disease. To associate the immune phenotypes of cases with high hypoxia, we divided our cohort into Hypoxia^HI^ and Hypoxia^LOW^ groups based on a top versus bottom quartile dichotomization (n=44 patients each). All subsequent analyses, unless stated otherwise, were performed using these groups. Clinical and pathological parameters for both groups are shown in **Supplementary Table 2**.

**Figure 2:**
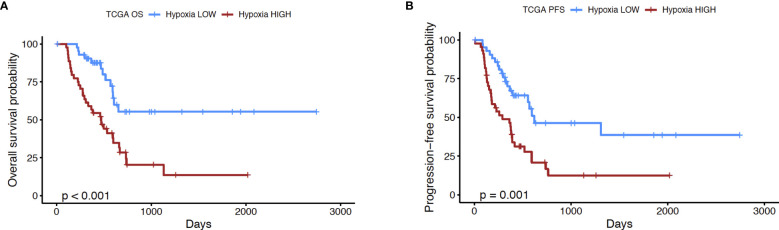
Prognostic analysis of hypoxia score. Kaplan-Meier plots displaying the **(A)** overall survival (OS) and **(B)** progression-free survival (PFS) of PDAC patients in Hypoxia^HI^ versus Hypoxia^LOW^ groups. The number of cases in each group and number at risk are shown in the tables below each plot. p value shown in the plots was derived using log rank test.

In order to further explore the utility of the hypoxia score for pancreatic cancer, we performed the following analyses. First, we used a microarray dataset of 45 PDAC tumors and paired adjacent non-malignant pancreatic tissue (GSE28735) (39), to show that the hypoxia score was significantly elevated (p<0.0001) in pancreatic tumors relative to the non-tumor tissue sample group (**Supplementary Figure 2**). This dataset was used as it contained transcriptomic profiles from PDAC biopsies and paired non-malignant pancreatic tissue, the latter of which are not available in the TCGA PDAC cohort. We also sought to study the expression of key genes from our signature in PDAC cells incubated in hypoxic conditions. As such, we examined the expression of 4 key hypoxia-inducible genes from the 10 “seed” genes that were used to define the Buffa hypoxia score (25), in PDAC cell lines incubated in hypoxic conditions (0.5% O_2_) for 24 hours. Using two well-known PDAC cell lines, PANC-1 and BxPC3 (44), we interrogated the expression of the genes Solute Carrier Family 2 Member 1 (*SLC2A1*), also known as *GLUT1*, Vascular Endothelial Growth Factor A (*VEGFA*), Carbonic Anhydrase IX (*CA9*), and Hexokinase 2 (*HK2*) as shown in **Supplementary Figure 3**. In PANC-1, all four genes were significantly induced in hypoxia at 24h compared to normoxic controls: *SLC2A1* (6.5-fold, p*=*0.007), *VEGFA* (2.9-fold, p*=* 0.01), *CA9* (7.6-fold, p*=* 0.002), and *HK2* (34-fold, p*=*0.008). Comparable results were also seen in the BxPC3 cell line relative to normoxic controls: thus, *SLC2A1* (17.8-fold, p*=*0.005), *VEGFA* (3.2-fold, p*=*0.03), *CA9* (73.4-fold, p*=*0.01), and *HK2* (9.9-fold, p*=*0.004) when compared with normoxia controls. Taken together, these results further support the validity of this hypoxia score in PDAC. The score was found on average to be significantly elevated in PDAC relative to non-malignant pancreas tissues, and that a subset of hypoxia-inducible genes that comprise the score show induction in PDAC cell lines in hypoxic conditions. Next, we investigated if the hypoxia score was associated with patient outcomes. We compared the survival of cases with low and high hypoxia (Hypoxia^HI^ vs Hypoxia^LOW^, see **Figure 1B**). It was observed that Hypoxia^HI^ patients demonstrated significantly lower OS (**Figure 2A**) and lower PFS (**Figure 2B**), compared to Hypoxia^LOW^ patients. Finally, we used the multivariate Cox Proportional Hazards model to assess the association between overall survival in the entire TCGA PDAC cohort (*n*=171 with available clinical information) and the hypoxia score as well as, clinical variables (Age, Sex, Stage and Grade). When examined in this multivariate regression analysis, we noted that only the hypoxia score was independently associated to OS in the TCGA cohort (**Supplementary Figure 4**). The association between the Buffa hypoxia score and reduced survival has been previously shown in PDAC (45). However, we demonstrated in a multivariate analysis that this hypoxia score is an independent prognostic variable in PDAC. As such, investigating Hypoxia^HI^ cases might reveal immunological mechanisms associated with disease progression in these patients.

### High hypoxia status is associated with multiple features of immunosuppression in PDAC

**Figure 3:**
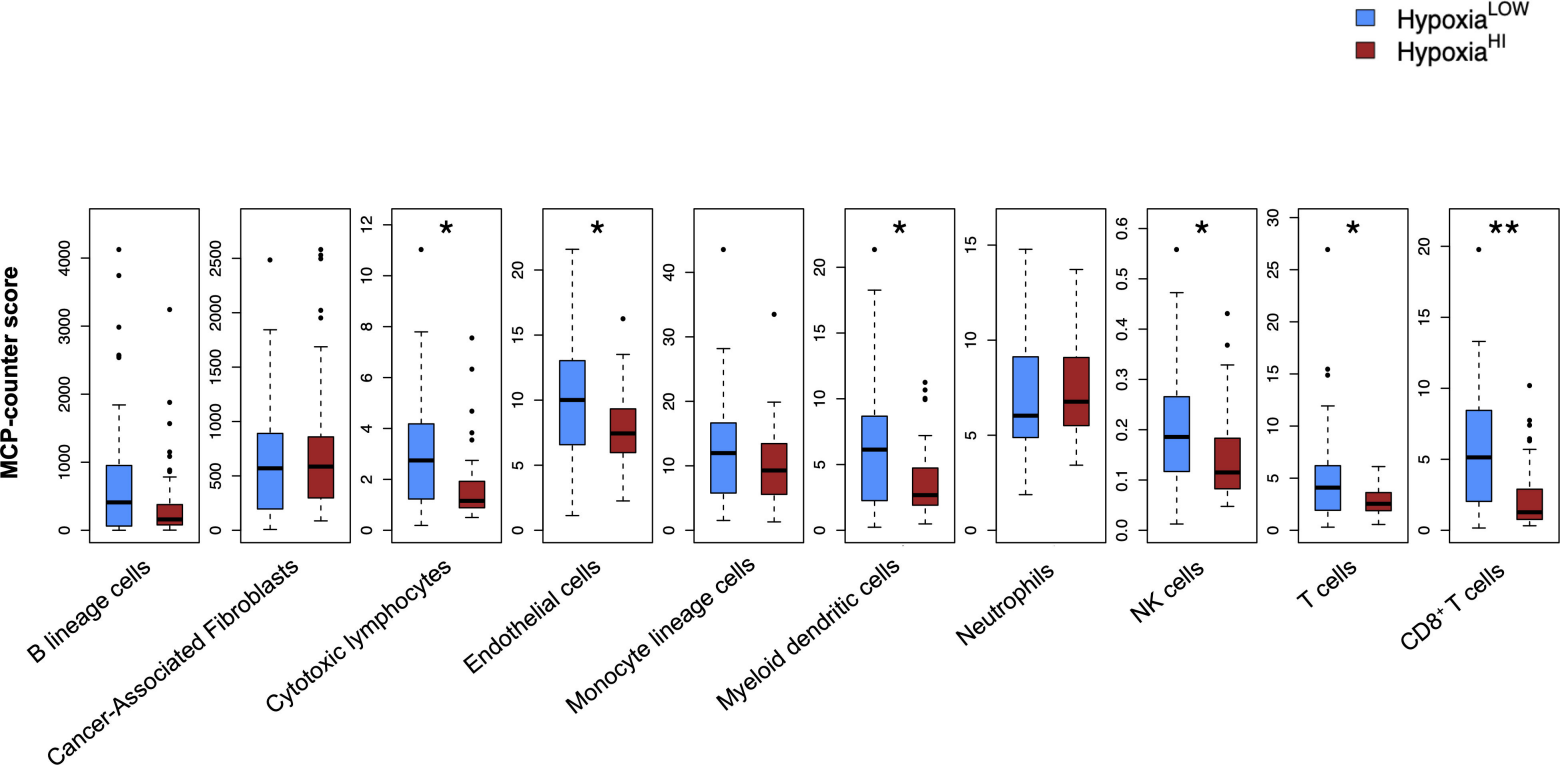
Profiling the TME of high versus low hypoxia scores. Box plots comparing 8 immune and 2 non-immune cell scores between Hypoxia^HI^ and Hypoxia^LOW^ groups. Cell abundance scores were computed using the MCP-counter algorithm. Statistical comparisons were performed through Mann-Whitney U test followed by Benjamini-Hochberg multiple testing correction. *p<0.05, ** p<0.01.

It is now well-established that hypoxia is a crucial mediator of immune escape, as well as of immunosuppressive signaling in the TME of solid organ cancers (16, 46). Immunohistochemical studies in patient biopsies of colorectal cancer have shown that the hypoxia marker CAIX is strongly expressed in “immune cold” tumors (47), which are marked by low or absence of T cell infiltrates and dendritic cells (DC) (48–50). As such, we performed analyses to dissect the immune microenvironment of Hypoxia^HI^ and Hypoxia^LOW^ cases. First, we used the MCP-counter method developed by Becht et al. for estimating cell-types in bulk tissue transcriptomes (36), to infer the abundance of 8 immune and 2 non-immune cell populations in both hypoxia groups. After correcting for multiple comparisons statistically, we found that the Hypoxia^HI^ group displayed notable differences in the abundance of key cell types in the TME, compared to the Hypoxia^LOW^ group (**Figure 3**). No statistically significant differences were observed for B lineage cells, CAFs, monocyte lineage cells and neutrophils between both hypoxia groups. In contrast, Hypoxia^HI^ tumors displayed markedly lower abundance scores for endothelial cells (p=0.02), myeloid dendritic cells (p=0.02), natural killer (NK) cells (p=0.02), total T cells (CD3^+^ T cells) (p=0.02), and CD8^+^ T cells (p=0.006) compared to Hypoxia^LOW^ cases. Compared to Hypoxia^LOW^ cases, the Hypoxia^HI^ group also displayed lower abundance of “cytotoxic lymphocytes”, a functionally defined signature meant to score for mRNA expression from both NK cells and T cells (36). Given the crucial role of DC in cancer immunity (51) and noting the difference between hypoxia groups in terms of myeloid DC scores, we sought to further explore this difference. Over the past 6 years, studies have shown that the conventional DC1 (cDC1) subset of DC, are critical for antigen cross-presentation and priming anti-tumor immunity (51). Using a previously defined gene signature for cDC1, we used singscore to compute cDC1 scores (**Supplementary Figure 5**). We demonstrate that Hypoxia^HI^ cases exhibit significantly lower cDC1 scores compared to Hypoxia^LOW^ cases. It is currently well-established that determining a tumor’s immunological phenotype depends not only on the presence of T cells but also the presence of important leukocytes such as NK cells and DC (50). We found that Hypoxia^HI^ cases displayed multiple features of a “cold” tumor with diminished anti-tumor immunity.

**Figure 4:**
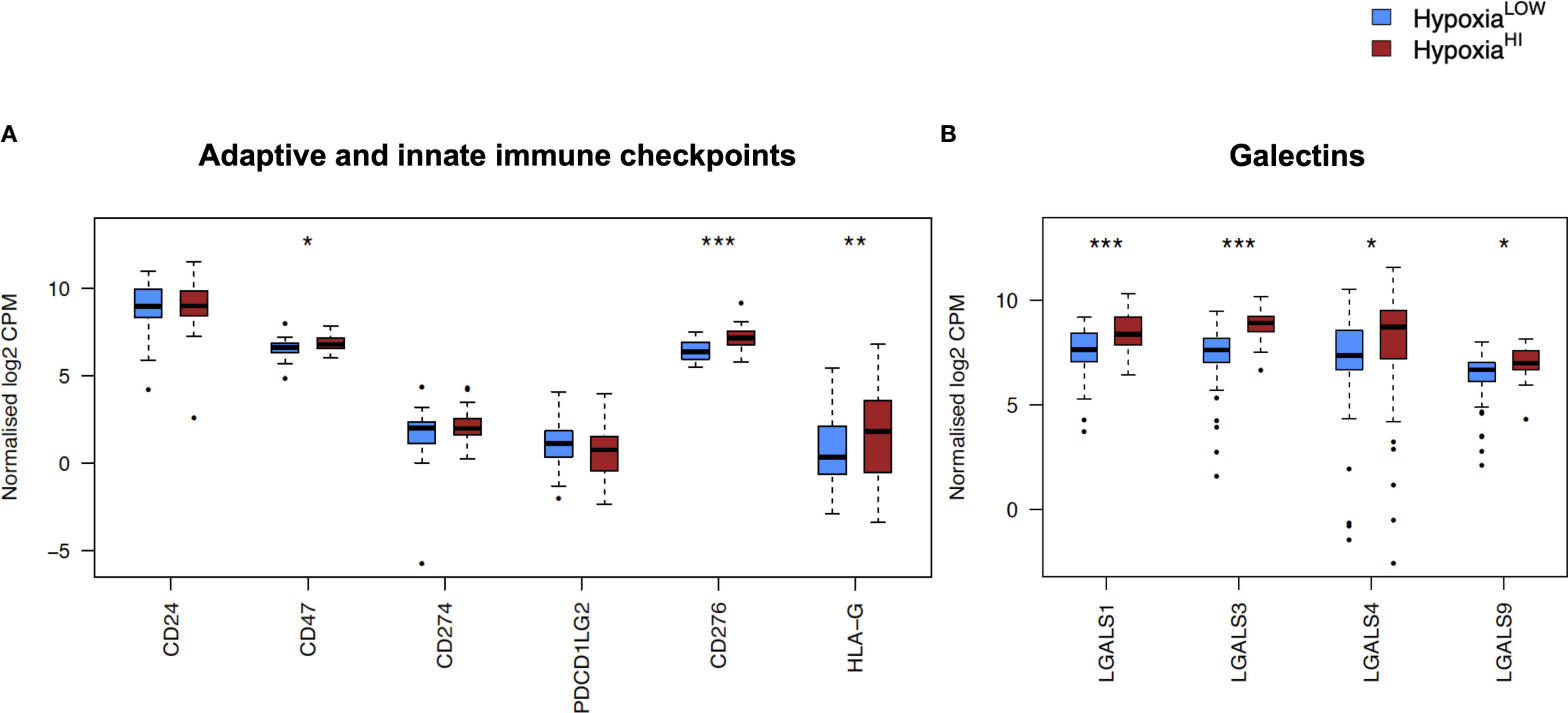
Comparisons of immune checkpoints and galectins gene expression profiles. Box plots comparing gene expression of **(A)** adaptive and innate immune checkpoint molecules and **(B)** selected galectin molecules with roles in immune escape. Gene expression is displayed as log2-transformed, normalized CPM (counts per million) values. Statistical significance shown on the plot represents FDR values from transcriptome-wide differential gene expression analysis between Hypoxia^HI^ and Hypoxia^LOW^ groups. *FDR<0.05, **FDR<0.01,***FDR<0.001.

**Figure 5:**
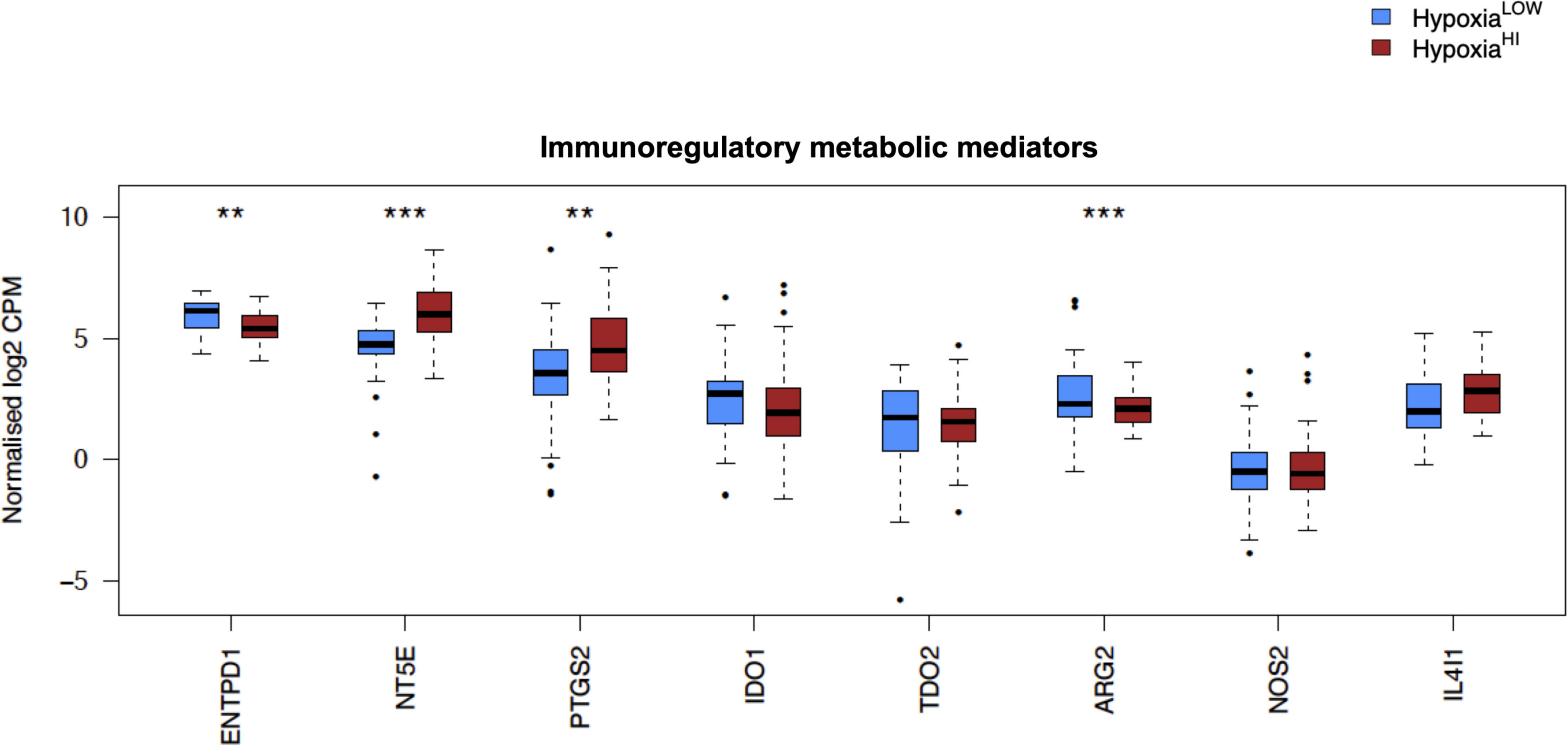
Comparisons of immunoregulatory metabolic mediators. Box plots comparing gene expression of enzymes purported to play a key role in promoting an immunosuppressive TME. Gene expression is displayed as log2-transformed, normalized CPM (counts per million) values. Statistical significance shown on the plot represents FDR values from transcriptome-wide differential gene expression analysis between Hypoxia^HI^ and Hypoxia^LOW^ groups. **FDR<0.01,***FDR<0.001.

In order to investigate the molecular mechanisms that might account for a “cold” tumor status in cases with high hypoxia scores, we performed differential gene expression profiling between Hypoxia^HI^ and Hypoxia^LOW^ cases and performed FDR corrections. Based on the results of the MCP-counter analyses, we compared the expression of selected immune-related genes coding for molecules known to negatively regulate anti-tumor immunity. First, we profiled key immune checkpoint molecules (which can be expressed both on tumor and non-tumor cells), that regulate both adaptive and innate immunity (52, 53). These include the “don’t-eat-me” signaling molecules which inhibit macrophage phagocytosis of tumor cells, *CD24* and *CD47*, the checkpoint molecules *CD274* (PD-L1), *PDCD1LG2* (PD-L2) and *CD276*, as well as the non-classical major histocompatibility complex I (MHC-I) molecule, *HLA-G* which inhibits NK cells (52, 53). As shown in **Figure 4A**, we observed a statistically significant increase in the expression of *CD47*, *CD276* and *HLA-G* but not of the other checkpoint molecules in Hypoxia^HI^ tumors relative to Hypoxia^LOW^. Second, we profiled genes encoding 3 of the best characterized members of the galectin family (Galectin-1, Galectin-3, and Galectin-9), which have roles in both cancer progression and immune modulation (54), as well as Galectin-4, which was recently shown to be associated with immune escape in PDAC and capable of inducing T cell apoptosis (55). Remarkably, expression of all four galectin genes *LGALS1*, *LGALS2*, *LGALS3* and *LGALS4* was observed to be significantly higher in Hypoxia^HI^ as compared to Hypoxia^LOW^ cases (**Figure 4B**). Third, we interrogated both groups for genes encoding key enzymes known to play critical roles in immunosuppression in the TME as shown in **Figure 5**. We analyzed expression of the ectonucleotidases CD39 (*ENTPD1*) and CD73 (*NT5E*) which mediate distinct steps in the conversion of extracellular ATP to adenosine, a potent inhibitory signal for immune cells (56), and the potential inflammatory mediator cyclooxygenase-2 (COX-2, *PTGS2*) (57). We also analyzed the expression of genes for 6 genetically unrelated amino acid catabolizing enzymes known to have immunosuppressive functions. As reviewed previously (58–60), the amino acids they catabolize are tryptophan (tryptophan 2,3-dioxygenase: *TDO2* as well as indoleamine 2,3-dioxygenase 1 and 2: *IDO1* and *IDO2*), arginine (inducible nitric oxide synthase: *NOS2* as well as arginase 1 and 2: *ARG1* and *ARG2*), and phenylalanine (Interleukin 4-induced 1: *IL4I1*) (58–60). It was observed that *ARG1* and *IDO2* mRNA expression levels in our TCGA PDAC cohort were below the abundance threshold applied in differential gene expression analysis, and therefore these genes were not included in subsequent analyses. When we compared both hypoxia groups, we noted that relative to Hypoxia^LOW^ tumors, Hypoxia^HI^ cases displayed lower expression for *ENTPD1* (CD39) and *ARG2* but significantly elevated expression of *NT5E* (CD73) and *PTGS2* (COX-2) (**Figure 5**). No differences were observed between groups for the expression of *IDO1*, *TDO2*, *NOS2* and *IL4I1*. Thus, these results demonstrate the activation of distinct metabolic pathways in cases with a high versus low hypoxia status. Taken together, our gene expression profiling demonstrated that Hypoxia^HI^ tumors displayed the upregulation of multiple molecules associated with an immunosuppressive TME. These included immune checkpoints, galectins and key metabolic mediators indicating that distinct pathways underlie the formation of an immunosuppressive TME in PDAC.

### Machine learning-based feature selection to predict high hypoxia status

**Figure 6:**
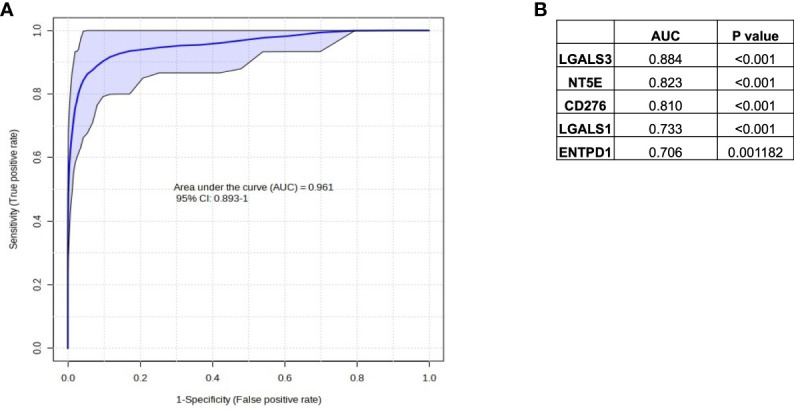
Selection of immune genes with high correlation to Hypoxia^HI^ status in TCGA. **(A)** ROC curve and AUC value for the top 5 gene features for classifying a case as Hypoxia^HI^. **(B)** Individual gene names and AUC values for each of the 5 gene features are provided in the table. Note that ENTPD1 exhibits an inverse relationship with hypoxia scores.

**Figure 7:**
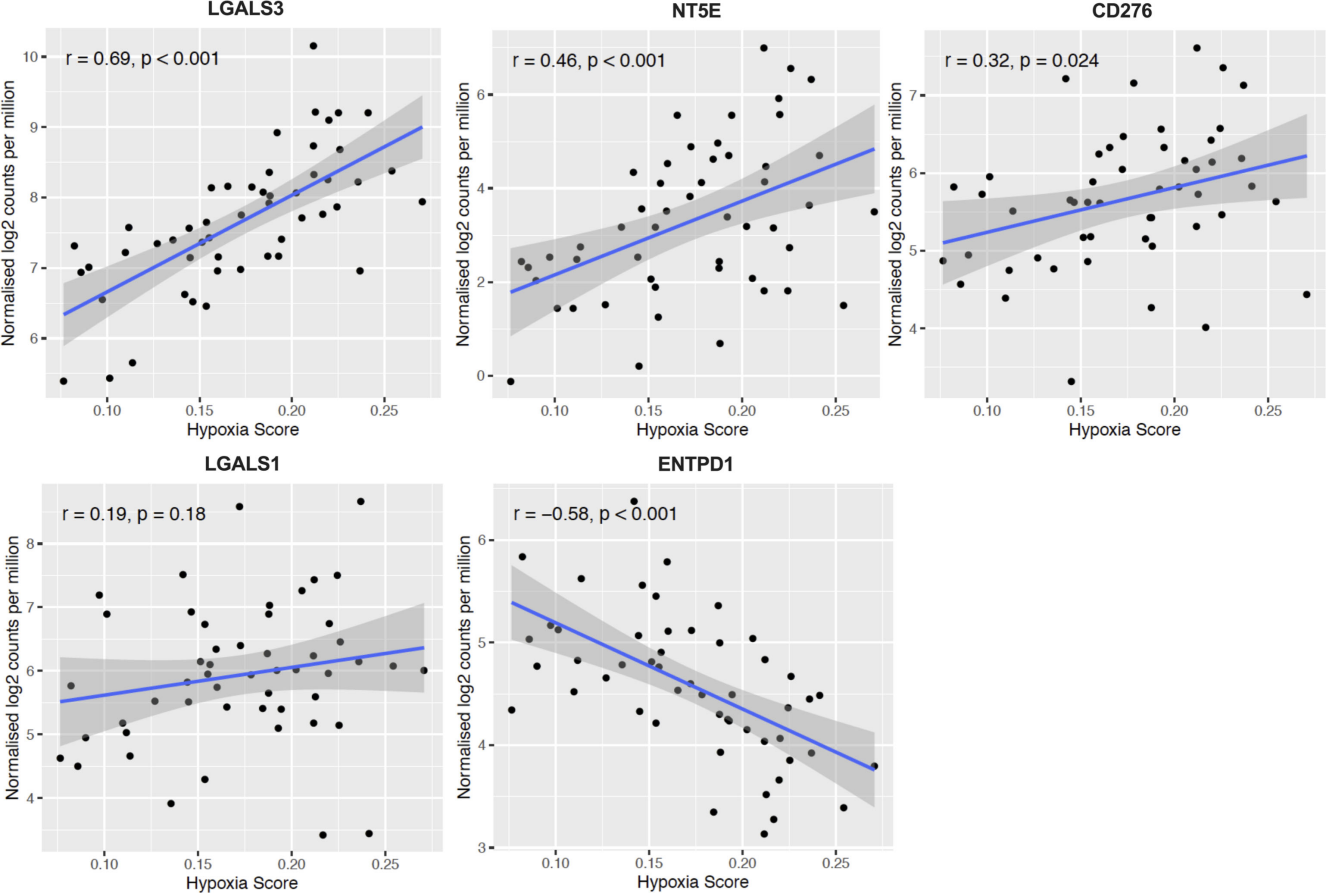
Confirmation of Hypoxia^HI^ correlated immune genes in additional cohort. Scatter plots depicting correlation between hypoxia score and the top 5 genes that could best classify a case as Hypoxia^HI^ in validation dataset of 51 PDAC cases (GSE79668). Spearman correlation coefficients r, and p values are plotted on the graphs. Each dot represents a single tumor sample.

**Figure 8:**
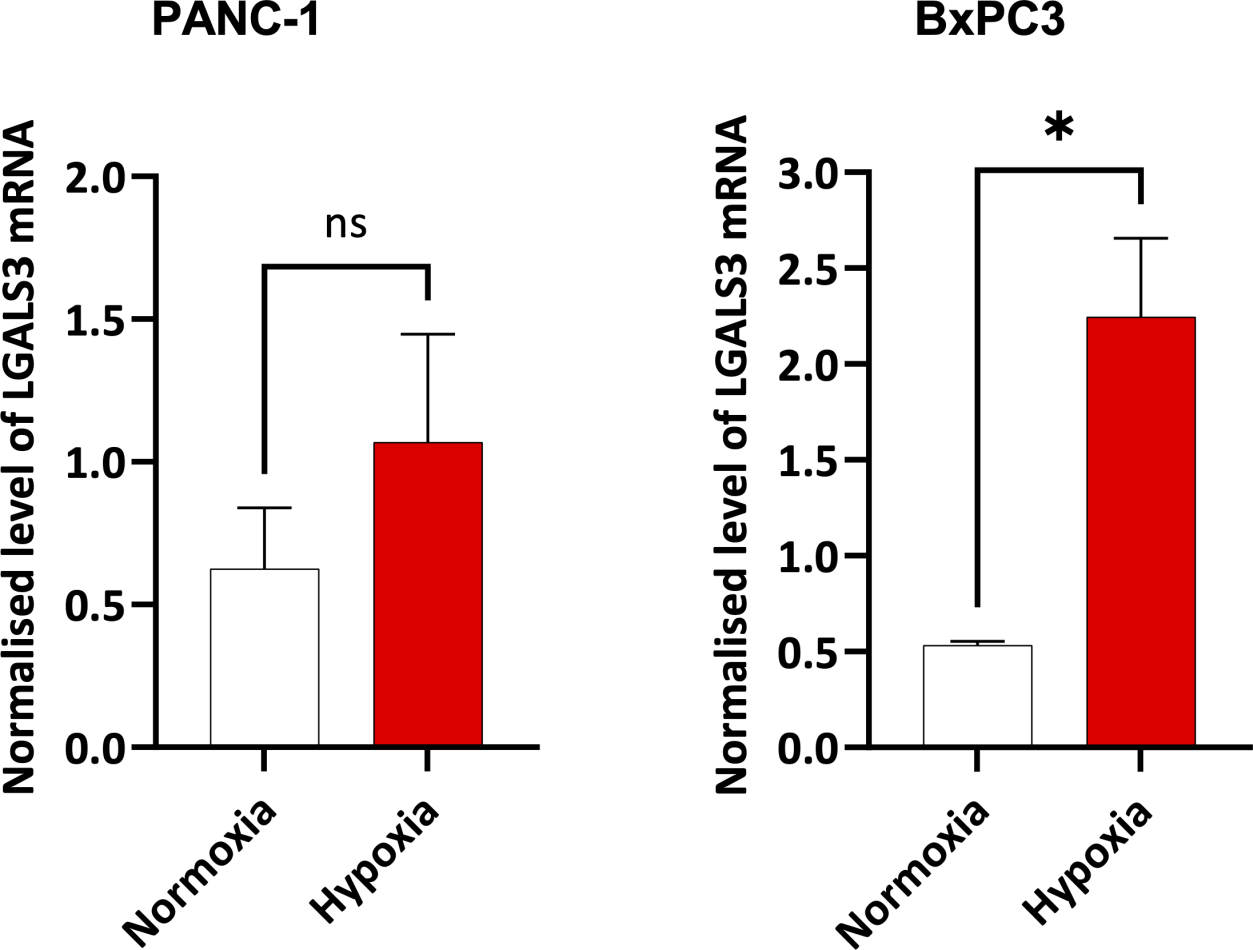
Expression of LGALS3 in hypoxic pancreatic cancer cells. Barplots showing normalized mRNA expression for LGALS3 in PANC-1 and BxPC3 pancreatic cancer cell lines incubated in hypoxia (0.5% O_2_) for 24 hours. mRNA expression was normalized to β2M. Data are from four independent experiments and are shown as means ± SEM. Paired t-test was used to assess the significance. *p<0.05. ns, not significant.

In **Figures 4** and **5**, we investigated 18 genes with known immunosuppressive functions to show that multiple, distinct genes are elevated in Hypoxia^HI^ tumors. Next, we sought to further explore which molecules were most strongly associated with a high hypoxia status. Using a random forest machine learning algorithm together with feature selection method (VarSelRF package using R) (61), to find a minimum set of genes that could predict Hypoxia^HI^ status. This method revealed 5 genes that could predict Hypoxia^HI^ and ROC (Receiver Operating Characteristic) analysis for these 5 genes displayed an AUC (Area Under ROC Curve) value of 0.961 (**Figure 6A**). The 5 genes, ranked by their AUC values are shown in **Figure 6B**, are *LGALS3*, *NT5E*, *CD276*, *LGALS1*, and *ENTPD1* (CD39). It is pertinent to note that *ENTPD1* displayed an inverse correlation with Hypoxia^HI^ status. To further assess the association of these genes with high hypoxia scores (i.e. Hypoxia^HI^) in PDAC, we performed Spearman correlation between expression levels of these genes and the hypoxia score in an additional cohort of RNA-seq data from 51 PDAC patients (GSE79668) (62). The expression of *LGALS3*, *CD276*, *NT5E* was positively correlated to hypoxia score expression in this cohort. The correlation coefficients and p values are shown in **Figure 7**. The gene *LGALS3* displayed the highest and most statistically significant positive correlation (r=0.68, p<0.0001) with hypoxia score expression. Similar to what was observed for the TCGA cohort, *ENTPD1*, also exhibited a negative significant correlation to the hypoxia score in this secondary cohort. *LGALS1* did not show a statistically significant positive or negative correlation with hypoxia score expression. Collectively, these findings suggest that immunomodulatory genes such as *LGALS3*, *CD276* and *NT5E* are the primary molecular signals that distinguish a highly hypoxic TME in PDAC cases. Given that *LGALS3* expression was observed to be strongly and positively correlated with the hypoxia score in both the TCGA cohort and the additional cohort, we interrogated the expression of *LGALS3* in PDAC cell lines in hypoxic conditions *in vitro*. As shown in **Figure 8**, *LGALS3* was significantly induced by 24h of hypoxia (0.5% O_2_) in BxPC3 cells (4.2-fold, p*=*0.02). While we did note a trend towards increased expression in PANC-1 cells, these results did not achieve statistical significance (1.7-fold, p*=*0.13).

## Discussion

### Hypoxia phenotyping in PDAC using gene signatures

Currently, PDAC remains one of the most treatment-refractory and clinically challenging cancers in clinical practice (2). A growing body of scientific literature now supports the idea that targeting tumor hypoxia in solid organ cancers can sensitize them to radiation, chemotherapy, and immunotherapies (15, 63, 64). Currently, hypoxic-tumor targeted therapies fall into four major categories: 1) Hypoxia-activated prodrugs that can kill tumor cells in hypoxic zones, 2) Molecules that inhibit HIF thereby abrogating HIF-downstream signaling, 3) Therapies that increase tumor oxygenation and 4) Drugs that modulate hypoxia-associated TME remodeling such as acidosis (e.g. CAIX) (15, 63, 65). Given the remarkable successes observed in certain tumor types (e.g. melanoma, renal cell carcinoma) using immune checkpoint blockade (9), identifying novel strategies for immune modulation of hypoxic pancreatic cancers is a critical priority. In this report, we utilized a well-established hypoxia signature to demonstrate that high hypoxia status is associated with distinct hallmarks of immunosuppression. Moreover, we utilized a machine-learning approach to identify *LGALS3* (Gal-3) as a potential therapeutic target for abolishing hypoxia associated suppression in PDAC (**Figures 6**–**8**). Using this innovative approach for further exploration of immunological profiles of PDAC tumors with high hypoxia gene expression, we identified potential therapeutic targets to improve immunotherapy responses in hypoxic cancers. However, *in vivo* models will be essential to further validate these observations.

Multiple reports have reported the derivation of a hypoxia signature as reviewed by Abou Khouzam et al. earlier this year (15). It is pertinent to note that the same group reported the derivation of an 8-gene hypoxia signature that showed prognostic value in PDAC as well as an association with “immune cold” TME (24). Direct measurements of oxygen partial pressure in limited clinical samples have revealed that pancreatic and prostate cancers are highly hypoxic (66). However, in the absence of direct measurements, hypoxia signatures have been developed and reported in the literature as useful scoring tools to assess hypoxia status in bulk tumor transcriptomes (67). As such, in published reports on hypoxia, cases are either stratified by the expression levels of a hypoxia gene signature or by a hypoxia risk score, where the expression of hypoxia-linked genes (selected for the score through survival regression analyses) is multiplied by their regression coefficients (15). In the past few months, another report by Ren et al. utilized a compact 15 gene version of Buffa signature to derive a 6 gene risk score in PDAC (22). It is pertinent to note that all published hypoxia gene signatures and risk scores have been shown to be correlated with poor prognosis in PDAC (15, 22). In multiple published reports, patients were classified as high or low based on a median expression cut-off (22–24). In our study, we sought to compare cases with notable differences in hypoxia status, and therefore used a cut-off of top versus bottom quartiles of expression for our hypoxia score, an approach that is used to classify patients in certain gene expression profiling studies (68, 69). We selected the Buffa hypoxia signature, a widely reported signature that was recently used to identify hypoxia-related proteins involved in metastasis in a study of 17 different carcinomas (27). We further expanded upon previous reports using this signature, to demonstrate its utility for profiling PDAC tissues. Using an additional dataset of PDAC transcriptomic profiles, we showed that the PDAC tumor group exhibited significantly higher hypoxia scores relative to paired non-malignant pancreatic tissues (**Supplementary Figure 2**). We also demonstrated that key hypoxia-inducible genes were elevated in hypoxic PDAC cell lines relative to normoxic controls (**Supplementary Figure 3**). As a final point, we observed that hypoxia scores were significantly elevated in higher grade tumors (**Figure 1B**). This is corroborated by previous reports in the literature. Using a 9 gene hypoxia risk score, Zhuang et al. recently also demonstrated that higher histological tumor grades displayed increased hypoxia risk scores (45). Finally, we demonstrated for the first time using both univariate and multivariate survival analyses, that the Buffa hypoxia score is an important prognostic indicator in PDAC. Hypoxia scores and hypoxia risk scores reported by others also confirm this independent prognostic association with patient outcomes (24, 45), thereby confirming the importance of studying hypoxia-related gene expression in pancreatic cancer.

### Deciphering the immune landscape in hypoxic PDAC cases

The mechanisms through which hypoxia shapes an immunosuppressive TME are complex, and reviewed elsewhere (64, 70, 71). In our study, we sought to dissect the distinct immunological mechanisms that could account for the “cold” tumor phenotype exhibited by Hypoxia^HI^ cases. We identified multiple features suggestive of an immunosuppressive TME, that were associated with high hypoxia status. We observed that Hypoxia^HI^ and Hypoxia^LOW^ could be discriminated by both cell-type deconvolution using MCP-counter (**Figure 3**) and immunosuppressive gene expression profiling (**Figures 4**, **5**). Using MCP-counter, we demonstrated multiple features representing an “immune cold” TME marked by a paucity of T and B lymphocytes, NK cells and myeloid DC (49). Notably, the inverse relationship between CD8^+^ T cells and hypoxia has also been reported in immunohistochemistry studies of colorectal, breast and ovarian cancers where CAIX is used as a marker for hypoxia (47, 72, 73). In fact, in a mouse melanoma model, treatment with a small molecule inhibitor of CAIX increased frequency of effector T_H_1 skewed CD4^+^ T cells and increased the response of these tumors to immune checkpoint therapy with anti-PD-1 and anti-CTLA-4 antibodies (74). The significant differences in abundance of CD8^+^ T cells between cases with high versus low hypoxia might also account for why we observed increased CD73 (*NT5E*) but reduced CD39 (*ENTPD1*) gene expression in Hypoxia^HI^ cases. It is important to note that both ectoenzymes are known to be expressed on a wide range of cells in the TME (56). CD39 is a marker for T cell exhaustion and even though it was recently reported to be induced in terminally exhausted CD8^+^ T cells by tumor hypoxia (75), there are other features in hypoxic TMEs such as abnormal tumor vasculature and increased fibrosis, which mediate T cell exclusion (70). Thus, the exclusion of T cells from highly hypoxic tumors is one possible explanation for the reduced gene expression of CD39 observed in Hypoxia^HI^ versus Hypoxia^LOW^ cases. This was confirmed by our MCP-counter analyses (**Figure 3**), which revealed that Hypoxia^HI^ cases have significantly lower abundance of total T cells, cytotoxic lymphocytes and CD8^+^ T cells.

According to previous findings, hypoxic regions produce signals that recruit multiple populations of myeloid cells such as MDSC, TAM and neutrophils (16, 64). We did not see any significant increase in the abundance scores for monocyte lineage cells or neutrophils (**Figure 3**). There is notable heterogeneity in the published literature on hypoxia gene signatures and their association with myeloid cell types (71). In the recent report by Abou Khouzam et al. (24), using an 8 gene hypoxia signature and the CIBERSORTx (76) deconvolution method in the TCGA PDAC cohort and an additional cohort, the authors found high hypoxia scores to be associated with diminished M2 macrophages in the TCGA dataset, and with an elevated abundance of M0, which purportedly accounts for gene expression signatures of undifferentiated macrophages that were not polarized into M1 or M2 *in vitro* (77). One further point that warrants consideration is that TAM and other myeloid cells are recruited into hypoxic niches, which also harbor necrotic zones (16, 78). Given that RNA from highly hypoxic and necrotic niches might undergo significant degradation (79, 80), bulk tumor transcriptomics data might not capture the myeloid diversity in highly hypoxic tumors. The absence of differences between major monocyte lineage cells between Hypoxia^HI^ and Hypoxia^LOW^ groups might also account for why we failed to see differences in expression of a number of amino-acid catabolizing enzymes which can be expressed in both non-immune cells and myeloid cells (59).

One notable phenotype of immunologically suppressed tumors that we observed was the interplay between NK cells, DC and COX-2, where we observed reduced signature scores for cDC1, lower MCP-counter abundance scores for myeloid DC and NK cells, as well as the increased gene expression of COX-2 (*PTGS2*) in Hypoxia^HI^ vs Hypoxia^LOW^ cases (**Figures 3**, **5** and **Supplementary Figure 5**). The induction of COX-2 expression in hypoxic conditions has been reported in both colon cancer and ovarian cancer cells (81, 82). Moreover, while COX-2 is reported to have tumor-intrinsic roles in the development and progression of PDAC in murine models (83), recent findings also demonstrated a key role for COX-2 in hindering anti-tumor immunity. The interplay between COX-2 and the NK cell-cDC1 axis was revealed in a landmark study using murine melanoma models (38). The authors showed that NK cells mediate the recruitment of the cDC1 subset, which are critical for anti-tumor immunity, and COX-2 mediated production of Prostaglandin E2 interferes with both NK cell chemokine production and cDC1 chemokine receptor expression. It is pertinent to note that COX-2 is over-expressed in multiple cancer types and influences the function of MDSC and lymphocytes as well as DC (57). An additional phenotype that we noted with MCP-counter, was a reduced abundance for endothelial cells in cases with high hypoxia scores. These results are supported by *in vivo* findings, where a recent study utilizing intravital fluorescence microscopy in a murine orthotopic pancreatic cancer model, revealed an inverse relationship between vascular density and the fraction of hypoxic cells (84). Thus, when cell-type deconvolution results with MCP-counter (**Figure 3**), support the premise that hypoxia mediates immune cell exclusion (70).

### Immunomodulatory gene expression correlated with increased hypoxia in PDAC

We sought to further explore immunosuppressive signaling in hypoxic PDAC tissues by examining differentially expressed genes related to immune function. We saw significantly increased expression of a number of important genes that have a critical role in immune escape such as *CD276* and *CD47* (**Figure 4A**). These findings are corroborated by previous reports, as *CD276* was reported to be associated with high hypoxia scores in the report by Abou Khouzam et al. (24), and it was shown in breast cancer cells, that HIF-1 directly induced *CD47* transcription (85). However, we identified a novel association between Gal-4 and our hypoxia score (**Figure 4B**). Gal-4 was previously shown to be highly expressed at the protein level in PDAC tissue, and Gal-4 knockout murine tumors exhibited notable elevation of CD4^+^ and CD8^+^ T cell infiltration (55). Indeed, we demonstrate that all examined members of the galectin family in our analyses were expressed at higher levels in Hypoxia^HI^ tumors relative to Hypoxia^LOW^. As reviewed recently, galectins are β-galactoside binding proteins that display important intracellular and extracellular functions and are secreted from cells through a non-canonical pathway (86). Galectins are purported to be involved in fibrotic and inflammatory diseases as well as cancer, and multiple galectin-targeted therapies are currently in clinical trials (54, 86). Our primary interest in profiling and comparing galectins was due to their roles in immunosuppression via direct T cell apoptosis but also as immune checkpoints (e.g. Gal-9 and TIM-3) (54, 86). Using a machine-learning recursive feature selection approach (61), we identified *LGALS3* as the top gene that could classify a PDAC tumor as Hypoxia^HI^ and confirmed the correlation between high hypoxia scores and *LGALS3* expression in an additional cohort of PDAC RNA-seq data. In PDAC, studies have shown that Gal-3 induces inflammatory cytokine expression in pancreatic stellate cells (activated PSCs constitute a portion of the CAF population in PDAC) (87). We demonstrated induction of *LGALS3* gene expression in hypoxic BxPC3 cells and a trend towards increased expression in PANC-1 cells albeit not reaching statistical significance (**Figure 8**). Our results are partially corroborated by an earlier study which showed *LGALS3* expression in PANC-1 cells at 24h and 48h (88). Moreover, in the same year, Gonnermann et al. demonstrated that the protein expression of Gal-3 was significantly higher in BxPC3 and other PDAC cell lines compared to PANC-1 in normoxic conditions (89). This report also showed that galectin-3 could inhibit the proliferation of Gamma-delta T cells, which are of interest due to their tumor-killing ability (89). Thus, our observations on elevated expression of *LGALS3* in PDAC tumors and PDAC cell lines, as well as the aforementioned report by Gonnerman et al. (89), highlight Gal-3 as a potential therapeutic target to reverse hypoxia-mediated immunosuppression.

Our study also had some limitations. First, the PAAD cohort in TCGA is comparatively small and therefore, comparing patients from the top and bottom quartile only yielded 44 cases per group. Third, while cell-type deconvolution approaches in bulk transcriptomics are more effective in generating abundance scores for cell types such as T cells, estimating the fractions of more heterogeneous cell types such as myeloid cells are challenging *in silico* (90). Finally, while multiple signatures for hypoxia have been reported in the literature (67, 71), these signatures warrant benchmarking in prospective studies where the hypoxia probe pimonidazole can be given to patients prior to operating (15), and RNA-seq can be performed to assess the correlation of each signature to levels of pimonidazole labeling *in situ*. However, we demonstrate multiple features of immunosuppression that delineate cases with high hypoxia score expression. Moreover, we described 5 genes associated with hypoxia high status, of which the top 3 (*LGALS3*, *CD276* and *NT5E*) have already been identified as targets of interest in cancer (56, 86, 91). Moreover, the novel association between Gal-4 and high hypoxia status also warrants further investigation to reveal the biological role of this protein in PDAC progression and its potential relevance as a drug target. We anticipate that future studies using newly emerging spatial transcriptomics technologies will be essential to decrypt the gene expression profiles in hypoxic versus normoxic regions of the TME and in order to identify potential drug targets for hypoxic cancers (92).

## Data availability statement

The original contributions presented in the study are included in the article/**Supplementary Material**. Further inquiries can be directed to the corresponding author.

## Ethics statement

This study utilized publicly available gene expression data from existing repositories and experiments performed in human cell lines. This study and all experiments performed within were reviewed and approved by the University Research Ethics Committee at Coventry University, UK.

## Author contributions

HS: Conceptualization, Data curation, Formal analysis, Funding acquisition, Investigation, Methodology, Project administration, Supervision, Validation, Visualization, Writing – original draft, Writing – review & editing. AA: Conceptualization, Formal analysis, Investigation, Methodology, Validation, Visualization, Writing – original draft, Writing – review & editing. HK: Formal analysis, Investigation, Methodology, Validation, Visualization, Writing – original draft, Writing – review & editing. TG: Data curation, Formal analysis, Investigation, Methodology, Writing – original draft, Writing – review & editing. RG: Conceptualization, Methodology, Validation, Visualization, Writing – original draft, Writing – review & editing. BB: Conceptualization, Formal analysis, Funding acquisition, Investigation, Methodology, Project administration, Resources, Supervision, Validation, Visualization, Writing – original draft, Writing – review & editing.
