# Supplementary material for: High hypoxia status in pancreatic cancer is associated with multiple hallmarks of an immunosuppressive tumor microenvironment
Source: Front Immunol. 2024 Mar 6;15:1360629. doi: 10.3389/fimmu.2024.1360629 (PMC10951397; doi:10.3389/fimmu.2024.1360629)
Supplement: Supplementary file 1 [file DataSheet_1.pdf]

Supplementary Material – Sadozai *et al.*
Supplementary Figure Legends

Supplementary Figure 1. Study outline.
A schematic depicting the outline of analyses performed to investigate the relationship between hypoxia and immune profile of PDAC tumours.

Supplementary Figure 2. Hypoxia scores are elevated in tumour versus normal pancreatic tissue.
Box plot comparing hypoxia scores in microarray transcriptomic profiles of pancreatic cases and paired adjacent normal (non-malignant) pancreatic tissue. This microarray dataset (GSE28735) contains 45 tumour and paired adjacent non-malignant tissues.

Supplementary Figure 3. Expression of selected Buffa genes in pancreatic cells under hypoxia.
Barplots showing normalized mRNA expression for the selected Buffa signature genes in PANC-1 and BxPC3 pancreatic cancer cell lines in under hypoxia (0.5% O_2) for 24 hours. mRNA expression was normalized to β2M. Data are from four independent experiments and are shown as means ± SEM. Paired T-test was used to assess the significance. *p<0.05, **p<0.001.

Supplementary Figure 4. Multivariate survival analysis for the hypoxia score.
Forest plot depicting hazard ratios and confidence intervals for overall survival of hypoxia score expression and clinical covariates, derived from Cox multivariate proportional hazards analysis in the TCGA PDAC cohort.

Supplementary Figure 5. cDC1 are diminished in hypoxic PDAC cases.
Box plot comparing cDC1 gene signature scores between Hypoxia^{HI} and Hypoxia^{LOW} groups in the TCGA cohort. Statistical analysis was performed using the Mann-Whitney U test. ***p<0.001

Supplementary Tables

Supplementary Table 1. Primer pairs for qPCR.

Gene name	Forward primer sequence (5'-3')	Reverse primer sequence (5'-3')
LGALS3	GCACCTCCTCGCCAGC	CCCAGGCAAAGGCAGGTTAT
SLC2A1	CAGGGAGCAGGAGACCAAAC	GGTGGACCCATGTCTGGTTG
VEGFA	CAGCGCAGCTACTGCCATCCAATCGAGA	GCTTGTCACATCTGCAAGTACGTTCGTTTA
CA9	TAGCCCTGGTTTTTGGCCTC	GTAGCTCACACCCCCTTTGG
HK2	ACGCCAAAATCACGTCTCC	AGAGAGGCGCATGTGGTAGA
β2M	GGCTATCCAGCGTACTCCAAAG	CAACTTCAATGTCGGATGGATG

Supplementary Table 2. Clinical and pathological features of Hypoxia^{LOW} and Hypoxia^{HI} cohorts.

Quantitative data are presented as group medians with interquartile range (IQR) in brackets. Categorical data are shown as cases and as a percentage of hypoxia cohorts in brackets. Statistical comparisons were performed using log-rank, Mann-Whitney U, and chi-square tests. Significant results are presented in bold.

Parameter	Hypoxia^{LOW} (n=44)	Hypoxia^{HI} (n=44)	*P* Value
**OS (days)**	674 (336-724)	480 (231-614)	**0.00037 (log rank test)**
**PFI (days)**	590 (276-602)	372 (129-466)	**0.001 (log rank test)**
**Sex**			0.67 (chi-squared test)
**Female**	18 (41%)	21 (48%)	
**Male**	26 (59%)	23 (52%)	
**Age (years)**	63 (56-70)	63 (52-75)	0.98 (Mann-Whitney U test)
**Grade**			**0.003831 (chi-squared test)**
**G1**	13 (30%)	1 (2%)	
**G2**	23 (52%)	27 (61%)	
**G3**	6 (14%)	15 (34%)	
**G4**	1 (2%)	1 (2%)	
**GX (not available)**	1 (2%)	0 (0%)	
**Stage**			0.21 (chi-squared test)
**Stage I**	1 (2%)	0 (0%)	
**Stage IA**	2 (5%)	1 (2%)	
**Stage IB**	7 (16%)	2 (5%)	
**Stage IIA**	4 (9%)	6 (14%)	
**Stage IIB**	26 (60%)	34 (77%)	
**Stage III**	1 (2%)	0 (0%)	
**Stage IV**	2 (5%)	0 (0%)	
**SX (not available)**	0 (0%)	1 (2%)	

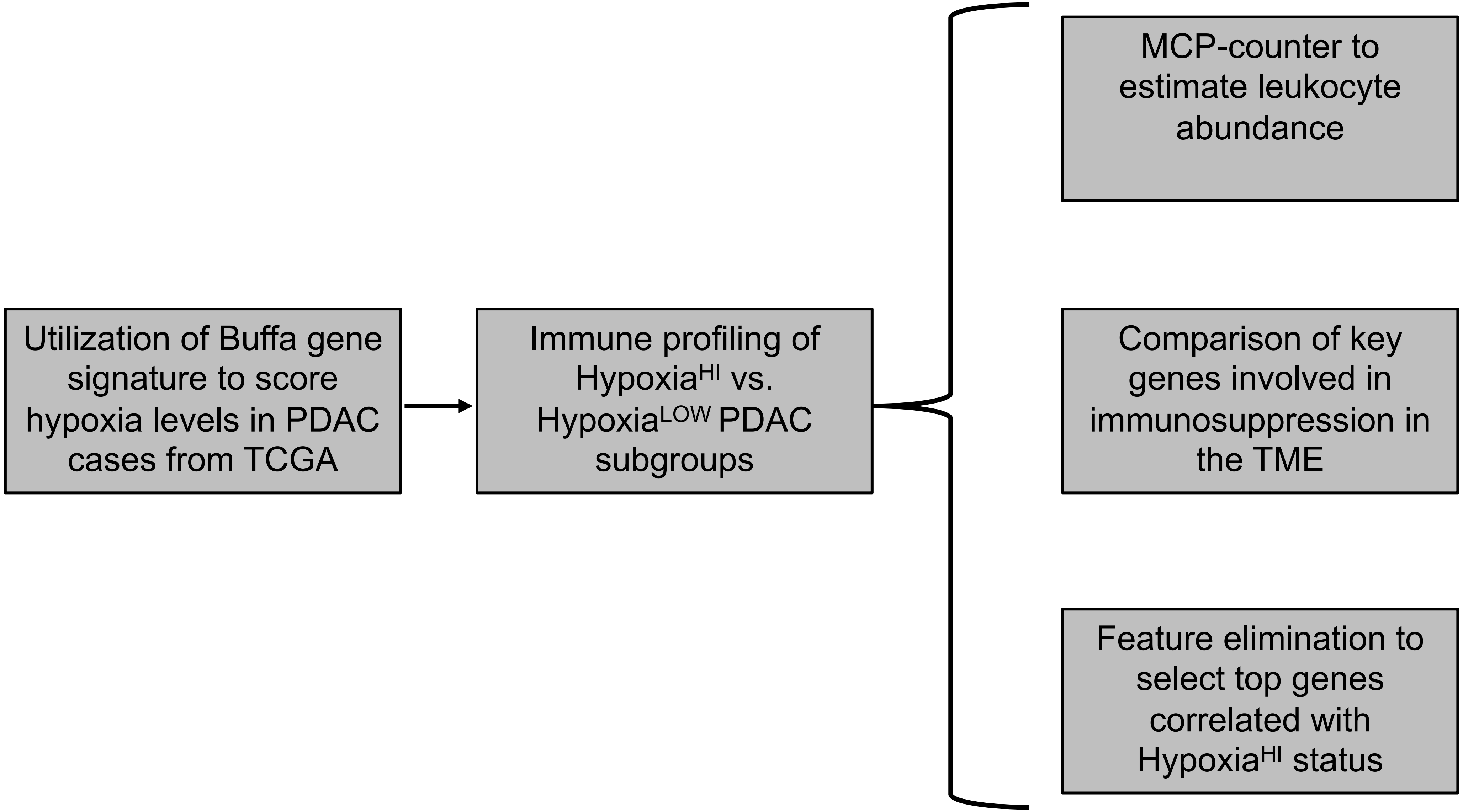

Supplementary Figure 1

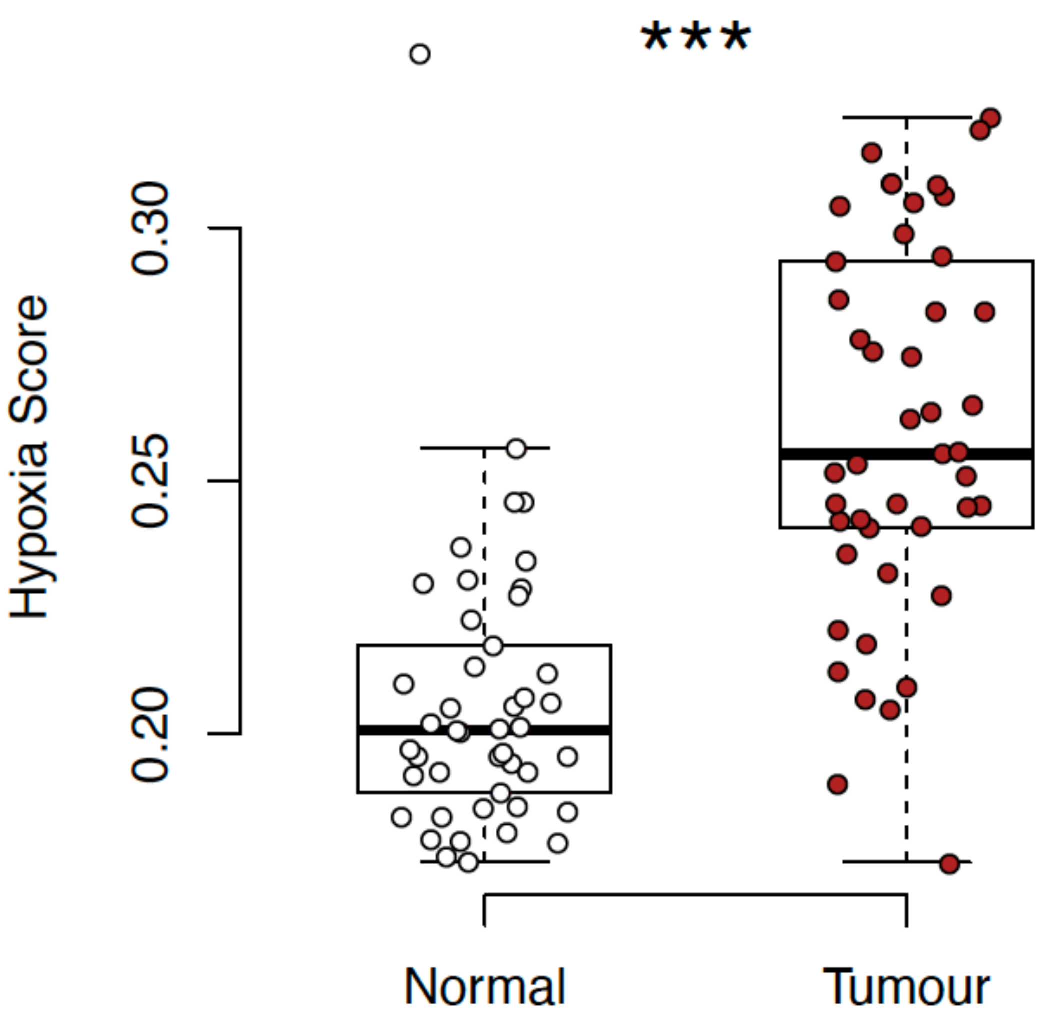

Supplementary Figure 2

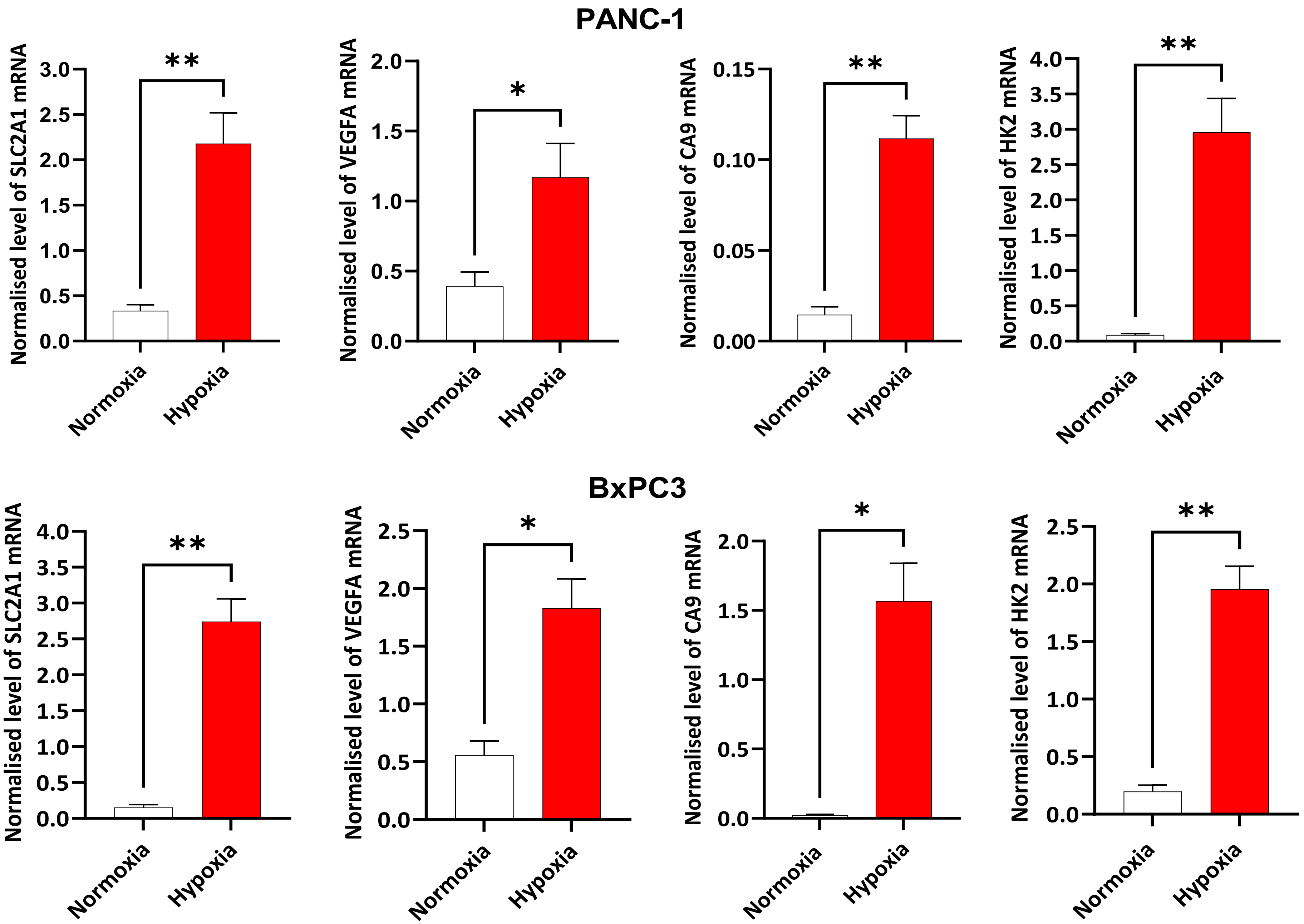

Supplementary Figure 3

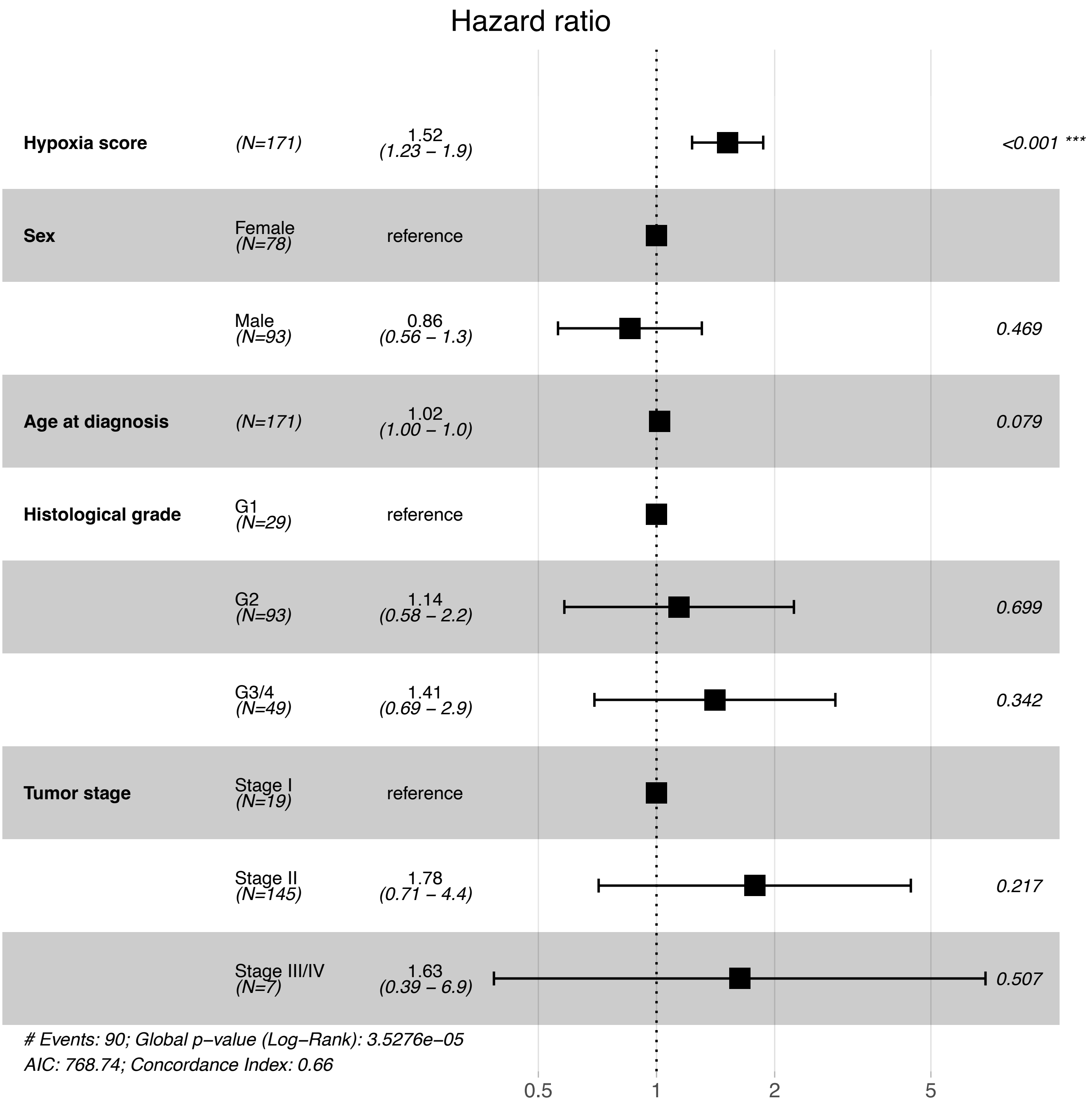

Supplementary Figure 4

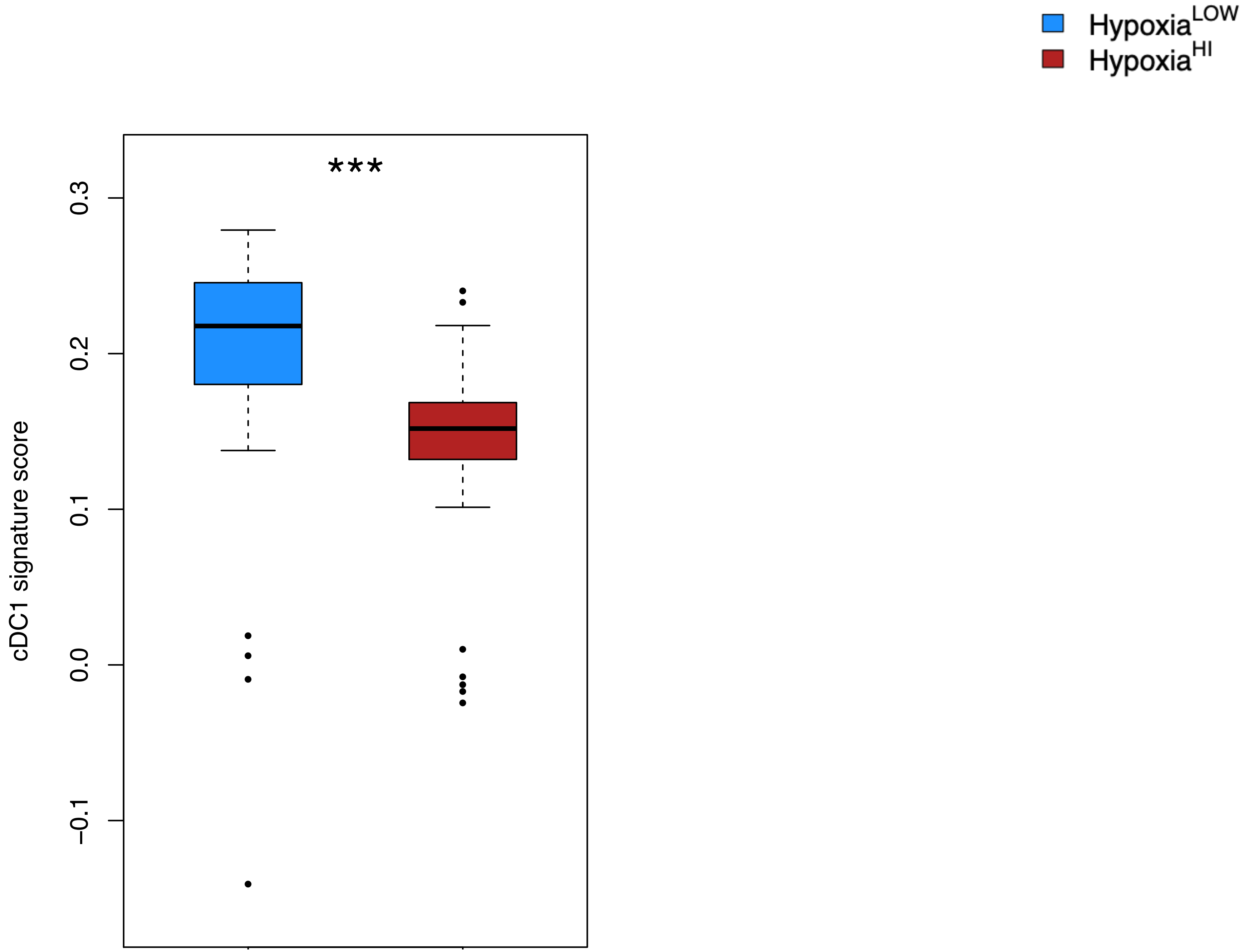

Supplementary Figure 5
